# Initial cardiac diagnostic imaging choices for obese patients: cost and outcomes with cardiac MRI

**DOI:** 10.1186/1532-429X-18-S1-Q27

**Published:** 2016-01-27

**Authors:** John Lisko, Nicholas C Boniface, Julianne Matthews, Brandon M Mikolich, J Ronald Mikolich

**Affiliations:** 1 Northeast Ohio Medical University (NEOMED), Rootstown, OH USA; 2 Sharon Regional Health System, Sharon, PA USA

## Background

Obesity is a significant risk factor for coronary artery disease. While nuclear myocardial perfusion imaging (MPI) and 2D Echo (2DE) are the most commonly used non-invasive imaging modalities in routine clinical practice, obesity often causes technical acquisition problems and artifact potentially leading to false positive results and further downstream testing. Cardiac MRI (CMR) image quality is not similarly limited by obesity. CMR is considered 2nd line for evaluation of chest pain, dyspnea, palpitations and syncope. This study was designed to assess the economic impact and clinical outcome of CMR for initial evaluation of cardiac symptoms in obese patients.

## Methods

Following IRB approval, the electronic medical records of a hospital-based Cardiology group practice were queried for new patients presenting with symptoms of chest pain, shortness of breath, palpitations or syncope. Each patient was followed for at least 1 year, after categorization by initial imaging modality, to quantify additional cardiac tests performed during the subsequent year and assess clinical outcome using death, stroke, myocardial infarction and malignant arrhythmia. Initial imaging decisions were at the discretion of the attending cardiologist. Patients who underwent initial CMR were compared to patients who had initial 2DE or MPI. Subsequent cardiac tests included in the analysis, along with their Medicare reimbursement were: EKG, Holter monitor, EP study, non-imaging stress, MPI, stress-echo, CMR, CT angiogram, cardiac catheterization, 2DE and TEE. Prevailing 2013 Medicare reimbursements were used for the analysis. The average economic cost to the healthcare system for patients having undergone initial CMR versus 2DE or MPI were computed and compared using independent sample t-tests. Adverse cardiac events over 1 year were computed for each group and also compared.

## Results

One hundred eighty-two patients met study inclusion criteria. 65 had an initial CMR, while 117 had initial 2DE or MPI. The average cost to the healthcare system per patient with an initial CMR for Medicare was $845.90 and $1,610.46 for private payors. The average cost to the healthcare system per patient with an initial 2DE or MPI for Medicare was $1,768.36 and $2,673.02 for private payors (see Figure). When compared, the cost difference between initial CMR versus initial 2DE or MPI was significant for both Medicare and private payors. (p=<.05)). Adverse cardiac event rates were non-significantly different for the 2 groups.

## Conclusions

After 1 year, obese patients who present with cardiac symptoms have a significantly higher cost of care if they had an initial 2DE or MPI, than those who had an initial CMR, with no difference in clinical outcome. With the advent of "Pay for Performance" policies, CMR provides a high quality, yet cost saving option for the inital cardiology evaluation of obese patients.

**Figure 1:**
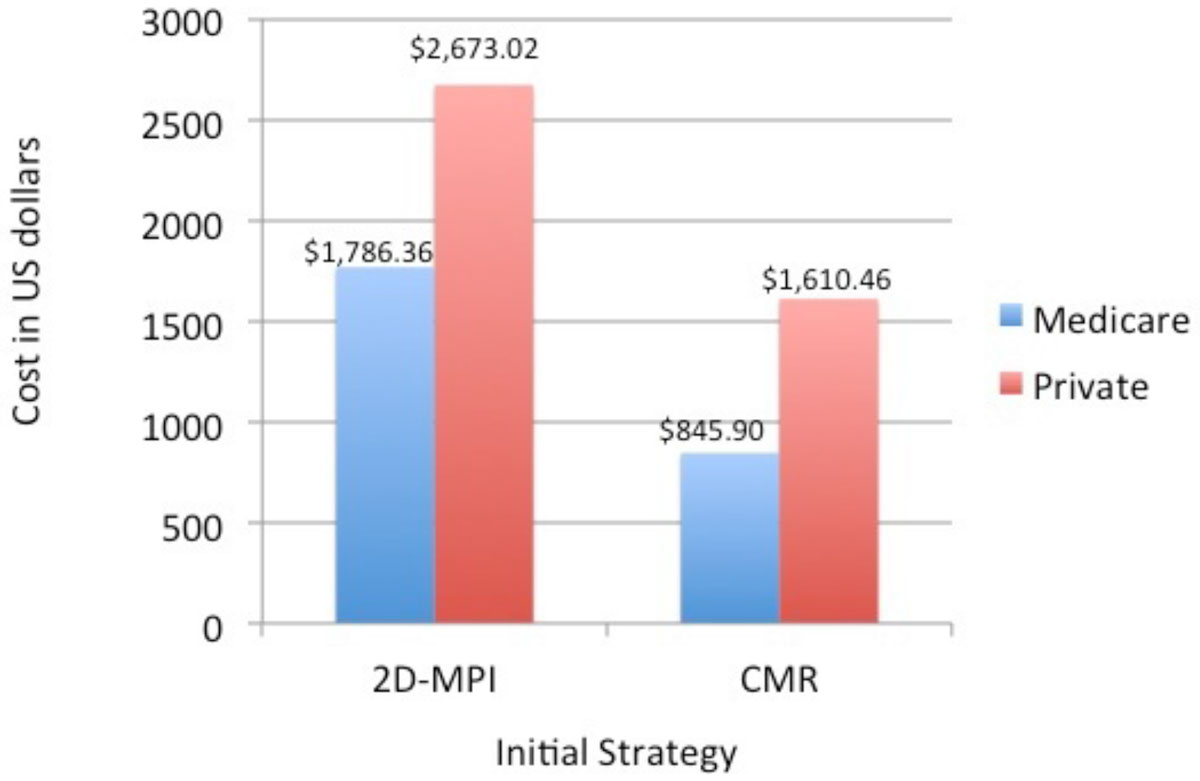
**One Year Mean Follow-Up Cost by Imaging Modality**.

